# A Probably Minor Role for Land-Applied Goat Manure in the Transmission of *Coxiella burnetii* to Humans in the 2007–2010 Dutch Q Fever Outbreak

**DOI:** 10.1371/journal.pone.0121355

**Published:** 2015-03-27

**Authors:** René van den Brom, Hendrik-Jan Roest, Arnout de Bruin, Daan Dercksen, Inge Santman-Berends, Wim van der Hoek, Annemiek Dinkla, Jelmer Vellema, Piet Vellema

**Affiliations:** 1 Department of Small Ruminant Health, GD Animal Health, Deventer, The Netherlands; 2 Department of Bacteriology and TSE’s, Central Veterinary Institute, part of Wageningen UR, Lelystad, The Netherlands; 3 Centre for Infectious Disease Control, National Institute for Public Health and the Environment, Bilthoven, The Netherlands; 4 Department of Epidemiology, GD Animal Health, Deventer, The Netherlands; 5 Delft University of Technology, The Netherlands; University of Texas Medical Branch, UNITED STATES

## Abstract

In 2007, Q fever started to become a major public health problem in the Netherlands, with small ruminants as most probable source. In order to reduce environmental contamination, control measures for manure were implemented because of the assumption that manure was highly contaminated with *Coxiella burnetii*. The aims of this study were 1) to clarify the role of *C*. *burnetii* contaminated manure from dairy goat farms in the transmission of *C*. *burnetii* to humans, 2) to assess the impact of manure storage on temperature profiles in dunghills, and 3) to calculate the decimal reduction time of the Nine Mile RSA 493 reference strain of *C*. *burnetii* under experimental conditions in different matrices. For these purposes, records on distribution of manure from case and control herds were mapped and a potential relation to incidences of human Q fever was investigated. Additionally, temperatures in two dunghills were measured and related to heat resistance of *C*. *burnetii*. Results of negative binomial regression showed no significant association between the incidence of human Q fever cases and the source of manure. Temperature measurements in the core and shell of dunghills on two farms were above 40°C for at least ten consecutive days which would result in a strong reduction of *C*. *burnetii* over time. Our findings indicate that there is no relationship between incidence of human Q fever and land applied manure from dairy goat farms with an abortion wave caused by *C*. *burnetii*. Temperature measurements in dunghills on two farms with *C*. *burnetii* shedding dairy goat herds further support the very limited role of goat manure as a transmission route during the Dutch human Q fever outbreak. It is very likely that the composting process within a dunghill will result in a clear reduction in the number of viable *C*. *burnetii*.

## Introduction

Q fever is a zoonotic disease caused by the obligate intracellular bacterium *Coxiella burnetii*. Domestic ruminants are considered to be the most important source of infection. In cattle, the disease is mainly asymptomatic [1], but in sheep and goats abortion, stillbirth and retention of foetal membranes can occur [2,3]. The bacterium is shed in urine, milk, faeces, and is found in high numbers in birth products of infected animals, causing environmental contamination. The main route of transmission of the bacterium to humans is by aerosols [2,4,5].

Until 2007, about twenty human Q fever cases were notified in the Netherlands annually [6]. Since then, Q fever started to become a major public health problem with 168, 1,000, and 2,357 notified human cases in 2007, 2008 and 2009, respectively [7]. These unprecedented annual outbreaks are largely explained by exposure of the general population to airborne *C*. *burnetii* contaminated dust particles originating from infected dairy goat herds with abortion storms [5, 8–12]. To reduce shedding, and thus environmental contamination, control measures were implemented, such as compulsory vaccination of all dairy sheep and dairy goats, and measures to reduce potential transmission, for instance by prohibiting removal of manure from stables within thirty days after lambing, and compulsory covering of manure after removal from the stable to reduce potential transmission [13,14].

These manure measures were implemented because of the assumption that manure played an important role in the transmission of *C*. *burnetii*. Not only urine and faeces [1], but especially birth products from infected small ruminants may contain large numbers of *C*. *burnetii*, leading to contamination of manure [15]. In several outbreaks, manure was suspected as the most probable source of the outbreak [16]. However, data confirming the contamination of manure by viable *C*. *burnetii* are lacking. In addition, no data are available that describe the anticipated reduction in the number of *C*. *burnetii* during storage, when composted. This is somewhat surprising as the manure control measures do have an impact on farm management and are implemented widely to avoid spread of *C*. *burnetii*.

The aims of this study were 1) to clarify the role of *C*. *burnetii* contaminated manure in the transmission of *C*. *burnetii* to humans, 2) to assess the impact of manure storage on temperature profiles in dunghills, and 3) to calculate the decimal reduction time of the Nine Mile RSA 493 reference strain of *C*. *burnetii* under experimental conditions in different matrices.

## Materials and Methods

### Mapping manure distribution patterns

In the Netherlands, farmers have to register transport of manure from their farm to its destination. Based on these records, distributions of manure from dairy goat farms with notified abortion waves caused by *C*. *burnetii* in 2008 and/or 2009 were compared with distributions of manure from a group of control farms. These control farms were defined as dairy goat farms without notified abortions caused by *C*. *burnetii*, which never had a positive PCR result in the mandatory bulk tank milk (BTM) surveillance program between its start in 2009 up to and including 2014, and which were BTM ELISA negative in 2008, before goats on these farms were vaccinated against *C*. *burnetii* [17]. Distribution of goat manure from both groups of farms in 2008 and 2009 was mapped. As a significantly higher incidence of Q fever patients has been demonstrated within a five km radius of an infected goat farm [5,8,10,12], all destinations of goat manure within a ten km radius of a herd with a notified abortion wave were excluded. The purpose of this exclusion is to preclude shedding by goats on infected farms as a possible source of environmental contamination. Manure destination areas from either case or control herds were identified by their four-digit postal code, of which there are more than 4000 in the Netherlands.

For all included four-digit postal code areas, destination and amount of manure, and incidence of human Q fever notifications in 2008 and 2009 were compared using descriptive statistics and negative binomial regression models (nbreg in STATA 13©). Human Q fever incidence was calculated for each four-digit postal code area by dividing the total number of Q fever patients in 2008 and 2009 by the number of residents present in the same area in 2009 based on Statistics Netherlands records [18]. In the negative binomial regression, the number of human cases per four-digit postal code area was included as dependent variable, and amount of manure or residents per four-digit postal code in 2009 were included as exposure. Independent variables that were included were whether manure originated from a case or control herd, and amounts of manure that were dropped (categorical in four categories).

### Participating farms

Owners of two dairy goat farms (farms A and B) with a history of *C*. *burnetii* related abortion waves, kindly gave permission to conduct this study on their farms. *C*. *burnetii* infection was confirmed by immunohistochemistry [3, 17]. Farm A had a herd size of 2,505 goats and farm B of 1,568 goats. On both farms, all goats were kept in deep litter stables all year round. At the start of the study, both farms were *C*. *burnetii* BTM PCR positive [17] in the Dutch BTM surveillance program, which became mandatory for all dairy sheep and dairy goat farms from October 2009 onwards [19]. Both farms were located in the province of Noord-Brabant, a province in the southern part of the Netherlands.

### Temperature measurements and manure sampling

Temperature development in manure was measured for 97 consecutive days after removal from the stable on the two farms. Upon removal of manure from the deep litter stables, dunghills were made on both farms. On farm A, the dunghill was 10 metres (m) long, 4.5 m wide and 3.5 m high. On farm B, the dunghill was 30 m long, 12.5 m wide and 7 m high.

**Fig 1:**
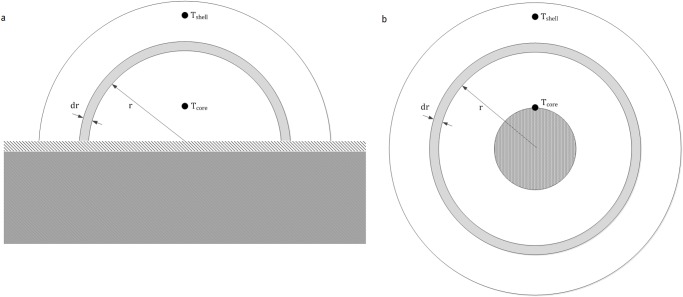
Schematic drawing of dunghill cross section (a). Schematic drawing of dunghill as simplified for setting up an energy balance (b). In Fig. 1a, a schematic drawing of dunghill cross section, placed on a concrete floor is presented. Please note that in reality the shape of a dunghill is less smooth. Calculations were performed with a height of the dunghill of 2.5 m, a width of 5 m, and a length (into the paper) of 10 m. These dimensions approach those of the dunghill of farm A. The measurement locations for shell and core temperatures are indicated. r = radius [m], T = temperature [°C]. In Fig. 1b, the dunghill was modelled as a hollow cylinder of infinite length with an inner radius of 0.5 m and an outer radius of 2.5 m. Temperature prediction was only possible between both temperature measurement locations (T_core_ and T_shell_). r = radius [m], T = temperature [°C].

Temperature measurements were carried out using a temperature measurement lance, fabricated and calibrated for this experiment by Peekel Instruments BV, Rotterdam, the Netherlands (www.peekel.nl). The calibrated temperature measuring equipment was connected to a computer to enable continuous temperature measurement. Data were stored using Signa Soft 6000 software. Temperature measurements inside the dunghills on both farms were performed at two locations as shown in Fig. 1a. The temperature of the core was measured at about 0.5 m from the concrete floor, while the shell temperature was taken at about 2.3 m from the concrete floor. Based on the results, an average daily temperature was determined for the core as well as for the shell of the dunghill.

On the day of removal of manure from the stables, manure samples were obtained in the deep litter stable on three different depths from the surface: 0–2 centimetre (cm), 18–20 cm, and 38–40 cm, respectively. A durable plastic polymer guide tube was used as a cylindrical pathway to the sampling sites, to collect manure samples on different levels in the dunghills. On both farms manure samples were obtained from the surface layer (0–20 cm), middle layer (90–100 cm) and deep layer (190–200 cm), respectively.

### Temperature profile estimates

In order to predict survival rates of *C*. *burnetii*, it is necessary to estimate the temperature profile between shell and core measurement locations. An energy balance was set up between both points, and for computational reasons, a simplification of the geometry of the dunghill was made as explained in Fig. 1b.

Setting up an energy balance on a slice of thickness ‘dr’ at radius r in the geometry shown in Fig. 1b gives:

$$\begin{array}{l} accumulation\;=\;in\;-\;out\;+\;production\\ 0=\underset{in}{\underset{︸}{\left(-2\pi rL\lambda \right){\frac{dT}{dr}|}_{r}\;}}-\;\underset{out}{\underset{︸}{\left(-2\pi rL\lambda \right){\frac{dT}{dr}|}_{r+dr}}}\;+\;\underset{production}{\underset{︸}{\left(2\pi rL\cdot dr\right){\dot{Q}}_{prod.}^{'''}}} \end{array}$$ (1)

with:

$\begin{array}{l} {\dot{Q}}_{prod}^{'''}=\text{rate of internal heat generation per unit volume}\left[\frac{W}{m^{3}}\right]\\ \lambda \;=\text{dung heat conductivity}\left[\frac{W}{m\cdot K}\right]\\ r\;=\text{radius}\left[m\right]\\ L\;=\text{dung hill lenght}\left[m\right]\\ T\;=\text{temperature}\left[{}^{\circ}C\right] \end{array}$

The first two terms of Equation 1 represent conductive heat transfer in the slice according to Fourier’s law of heat conduction [20]. In the third term, heat production inside the slice is described. Rewriting Equation 1 and solving the resulting differential equation gives the following result:

T(r)=Tcore+Q˙prod'''4λ(rcore2−r2)+Tshell−Tcore+Q˙prod'''4λ(rshell2−rcore2)ln(rshellrcore)ln(rrcore) (2)

Equation 2 shows how the temperature inside the dunghill varies with its radius. This equation is only valid for *r*_*core*_ *≤ r ≤r*_*shell*_. It was assumed that the rate of internal heat generation per unit volume (${\dot{Q}}_{prod}^{'''}$) does not depend on radius. In Equation 2, temperatures of core and shell (*T*_*core*_ and *T*_*shell*_), as well as the radius of core and shell (*r*_*core*_ and *r*_*shell*_) are known. If ${\dot{Q}}_{prod}^{'''}$ and the dung heat conductivity (*λ*) are also known, the temperature profile inside the dunghill can be calculated. Since these two parameters were not measured in the experiment, they need to be estimated. Looking at the terms in Equation 2, which contain ${\dot{Q}}_{prod}^{'''}$, it can be seen that for *r > r*_*core*_ these terms would always be positive, meaning that they would increase the temperature at every value for r. Therefore, more conservative temperature estimates would be obtained by setting ${\dot{Q}}_{prod}^{'''}$ to zero in Equation 2, neglecting internal heat generation altogether. Equation 2 then simplifies to Equation 3 (Mills, 1999):

T(r)=Tcore+Tshell−Tcoreln(rshellrcore)ln(rrcore) (3)

Using Equation 3, a temperature profile could be calculated for each of the 97 days for which measurements were available for farm A. For this purpose, the dunghill was divided into 25 parts with a thickness of 10 cm and a length L, analogous in shape to the segmented part with thickness dr in Fig. 1a. For all the segmented parts with a radius between *r*_*core*_ and *r*_*shell*_, the temperature at each day in the middle of each segmented part was calculated using Equation 3.

### Extrapolation of decimal reduction time from literature

The decimal reduction time of *C*. *burnetii* in milk was measured by Enright et al. [21] for temperatures between 143 (61.7°C) and 162 (72.2°C) degrees Fahrenheit. These data were fitted to the following Equation (4):

l10og(t)=−0.2258T+17.3307t=time[s]T=temperature[°C] (4)

Using extrapolation below 61.7°C (143°F), Equation 4 was used in combination with the results from the temperature profile calculations in order to predict whether or not *C*. *burnetii* in a certain segmented part survived 97 days in the dunghill at Farm A.

### *Coxiella burnetii* PCR in manure

Procedures for manure sample processing, DNA extraction, and qPCR detection of *C*. *burnetii* DNA have been described previously [22, 23]. Samples were scored as undetermined when no signals were observed for both *C*. *burnetii* and the internal control targets, indicating severe qPCR inhibition. In DNA extraction procedures, especially from complex environmental samples, many substances are co-extracted, which may interfere DNA amplification during qPCR. This can result in underestimations of the presence of DNA from a potential pathogen. To be able to estimate the number of *C*. *burnetii* organisms, differences between Cq values for internal control target *cry1*, obtained from samples and positive controls (p.c), were corrected for qPCR inhibition effects by using the following formula: ΔCq_*cry1*_ = Cq_*cry1* sample-_Cq_*cry1* p.c_.

Values for Cq_*cry1* sample_ and Cq_*cry1* p.c_ resemble Cq values obtained from samples and positive controls, respectively. The value of ΔCq_*cry1*_ is a measure for qPCR inhibition in a particular sample. This value is subtracted from the Cq values for *C*. *burnetii* targets *IS1111* and *com1*, to correct for qPCR inhibition effects.

An important assumption using this procedure is that all targets are affected by qPCR inhibition in the same order of magnitude. We estimated the number of *C*. *burnetii* organisms present per gram manure, based on Cq values for target *com1*, and using a DNA standard for *C*. *burnetii* (Vircell (www.vircell.com), cat. Nr.MBC018).

### Culture of *Coxiella burnetii* in naïve and spiked goat manure samples

To isolate *C*. *burnetii* from manure, 2 mL of manure was suspended in 10 mL of phosphate buffered saline (M: 0.01; pH: 7.2) and shaken for 10 minutes. The suspension was centrifuged for 10 minutes at 100 g. Supernatant was filtered stepwise over filters with pore sizes of 1.2 μm and 0.45 μm (Pall Cooperation, USA). Filtered material was centrifuged for 5 minutes, 15,000 g twice and the pellet was first suspended in 1 mL of culture medium without antibiotics (Eagle’s minimal essential medium (EMEM) with 10% bovine serum albumin, 1% non-essential amino acids (NEAA), 1% glutamax) followed by resuspension in 100 μL of culture medium. This suspension was inoculated onto a culture of Buffalo Green Monkey (BGM) cells and incubated for 14 days at 37°C in a closed flask as reported earlier [15]. Growth of *C*. *burnetii* was monitored by checking vacuolization of the BGM cells and confirmed by immunofluorescence staining, with the Nine Mile RSA 493 reference strain as positive control [15]. To evaluate the ability to isolate and culture *C*. *burnetii* from manure (positive control experiment), a spiking experiment was set up: 1 to 1.5 gram *C*. *burnetii* PCR negative goat manure was suspended in 2 mL PBS. To eliminate contaminating flora, the suspension was heated for 30 min at 99°C. After cooling down, 8.68 x 10^9^ *C*. *burnetii* Nine Mile strain bacteria were added. The number of bacteria was quantified according to Roest et al. [15].

### Calculated decimal reduction time

For the determination of the decimal reduction time (DRT) of *C*. *burnetii*, the Nine Mile RSA 493 reference strain was used in a concentration of 1 x 10^5^ bacteria per mL. The DRT was determined in PBS, PBS with 1.8 w/v% urea, PBS with 1.8 w/v% ammonia and in goat manure extract (9.5 gram of goat manure in 28.5 mL PBS). To determine the concentration of *C*. *burnetii* in the suspension before and after time-temperature treatment, ten-fold dilutions of the samples were made and inoculated on BGM cells. Cells were incubated for 14 days as described above. Growth of *C*. *burnetii* was monitored by PCR of the supernatant and finally by immunofluorescent staining [15]. The different *C*. *burnetii* solutions were treated using the following time-temperature combinations: 5, 10 and 15 seconds with 70 and 72°C, and 3, 6 and 9 min with 60 and 65°C. Immediately after treatment, samples were cooled down to room temperature. Samples with PBS-urea, PBS-ammonia and goat manure extract were washed twice at 10 minutes of centrifuging at 14,000g and resuspension in 1 mL of PBS before inoculation onto BGM cells. All measurements were done in triplicate.

The DRT at a certain temperature can be calculated using the formula:

DRT=t2−t1L10OG([start][end])

[24] with *t*_2_—*t*_1_ = the duration of treatment in which the change in concentration took place, and L10OG([start][end]) = the decimal reduction of the starting concentration to the concentration at the end. In this experiment, DRT was calculated as the average over three measurements over three time intervals per matrix at temperatures 60, 65, 70 and 72°C. The DRT in the matrix at other temperatures was extrapolated from DRT-temperature curve.

The data that were used for this study are freely available upon request according to the data sharing policies of PLOS ONE. The data could not be uploaded in a public data deposition because the data are owned by three different institutes that want to be informed when the data are used for other purposes than for this study. Requests can be directed to the small ruminant department of GD Animal Health in the Netherlands.

## Results

### Distribution of manure

**Fig 2:**
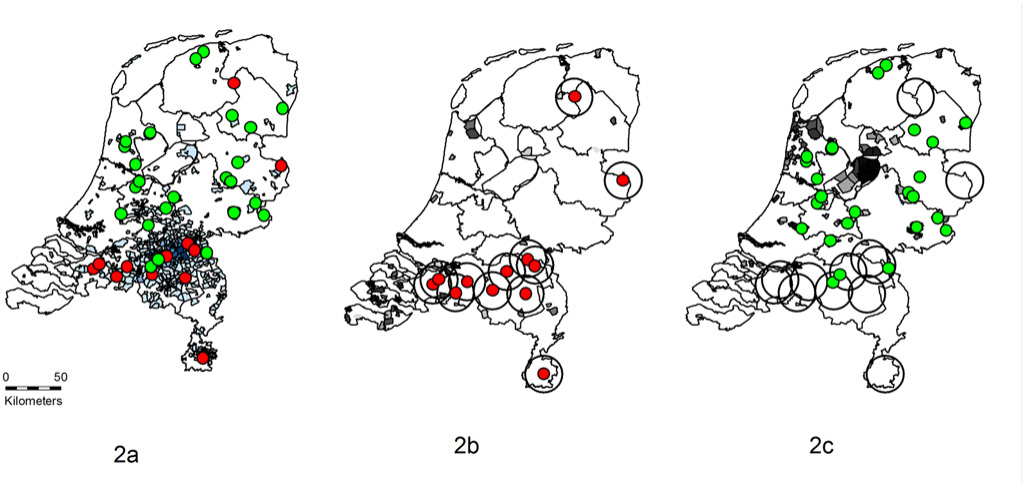
Distribution of manure and incidences of human Q fever patients. In Fig. 2a, twelve dairy goat farms with abortion waves caused by *Coxiella burnetii* in 2008 and/or 2009 (case farms; red dots), and 24 dairy goat farms without notified abortion waves caused by *C*. *burnetii*, bulk tank milk (BTM) PCR negative results between 2009 and 2014, and BTM ELISA negative results in 2008 from which records of manure distribution were available (controls; green dots), as well as incidences (number of cases per 100,000 residents) of human Q fever patients (the darker area, the more human Q fever patients) are presented. In Fig. 2b, distributions of manure from case farms outside a radius of ten km around case farms to four-digit postcode areas (dark colored) are presented. In Fig. 2c, distributions of manure from control farms outside a radius of ten km around case farms to four-digit (dark colored) are presented.

In 2008 and 2009, records of all 3,357 notified human Q fever patients were available. Incidence of human Q fever patients is presented per four-digit postal code area in Fig. 2a. In the same period, *C*. *burnetii* induced abortion waves were confirmed on twelve dairy goat farms (case herds). From these case herds, manure was removed 692 times in 2008 and 2009. This manure was distributed over 94 out of 3,972 four-digit postal code areas, and per area in which manure was distributed a median of 99,230 kg manure was distributed (25% percentile: 47,720–75% percentile: 202,540).

From 24 control herds, manure was removed 861 times in 2008 and 2009. This manure was distributed over 107 four-digit postal code areas. Per postal code area a median of 80,240 kg manure was distributed (25% percentile: 36,100–75% percentile: 199,260). After removal of the manure distributions in the 10 kilometre four-digit postal code areas around case herds, manure distribution of case herds remained in 54 postal code areas, and manure distribution of control herds remained in 103 postal code areas (Figs. 2b and 2c).

In 54 four-digit postal code areas in which manure from case herds was distributed, there were on average 5.1 human Q-fever cases per 100,000 residents (median 0; 25% percentile: 0–75% percentile: 0), in 2008 and 2009. In 103 postal code areas in which manure from control farms was dropped, there were on average 3.6 human cases per 100,000 residents (median 0; 25% percentile: 0–75% percentile: 0), in 2008 and 2009. In comparison, on average 99.8 human cases per 100,000 residents (median 9.3; 25% percentile: 0–75% percentile: 77.9) were found within a radius of ten kilometre around case farms, in 2008 and 2009.

Results of negative binomial regression showed no significant association between the incidence of human Q fever cases and the origin of manure (*P*-value 0.95). We also found no association with the amount of manure that was distributed and an interaction between case or control farms, and the amount of manure also tested non-significantly (*P*-value 0.81). In addition, to improve the precision of our results, we varied the time period that was included (from January 1^st^ 2008 until December 31^th^ 2010, and from the moment that an abortion wave occurred until six months after this event) but all models showed non-significant results.

### Temperature measurements and manure sampling

**Fig 3:**
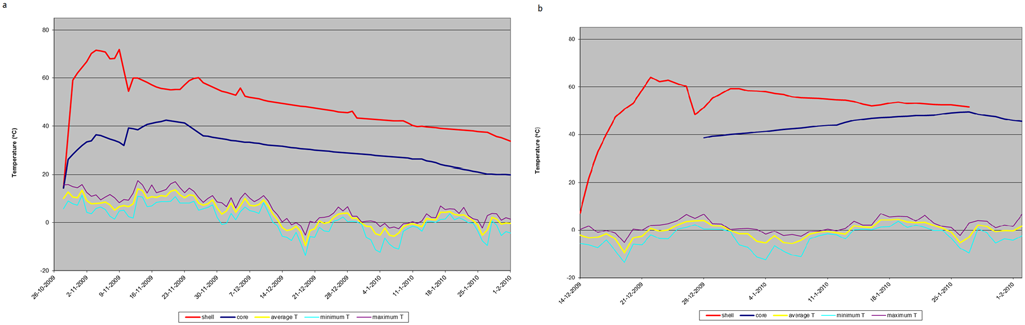
Outside and dunghill temperatures during the experiment. In Fig 3a, temperatures in the core (dark blue) and shell (red) of the dunghill on farm A are presented. In Fig 3b, temperatures in the core (dark blue) and shell (red) of the dunghill on farm B are presented. For both farms, the average (yellow), the minimum (turquoise) and the maximum (purple) outside air temperature in Eindhoven, the Netherlands (www.knmi.nl) during the experiments are shown. All temperatures are in degree Celsius.

In the shell of the dunghill on farm A, the highest temperature of 72°C was measured within four days after the start of the measurements. A shell temperature above 60°C was measured for twelve consecutive days. The temperature in the core rose less quickly and reached a temperature above 40°C for ten consecutive days (Fig. 3a).

In the shell of the dunghill on farm B, the highest temperature of 64°C was measured within five days after the start of the measurements. A shell temperature above 60°C was measured for five consecutive days. The temperature in the core of the dunghill on Farm B also rose less quickly than on farm A and reached a temperature above 50°C for ten consecutive days (Fig. 3b).

### Temperature profile estimates

Since temperature data for farm B were incomplete, caused by a technical problem, temperature profiles were only calculated for farm A. On this farm, the dunghill was 10 m long, 4.5 m wide and 3.5 m high at the start of the measurements. Temperature profiles were calculated using a height of 2.5 m since during the experiment the dunghill size settled to this height. The dunghill width used for the calculations was 5 m. Calculations were based on 97 consecutive days, starting on 28^th^ October 2009.

**Fig 4:**
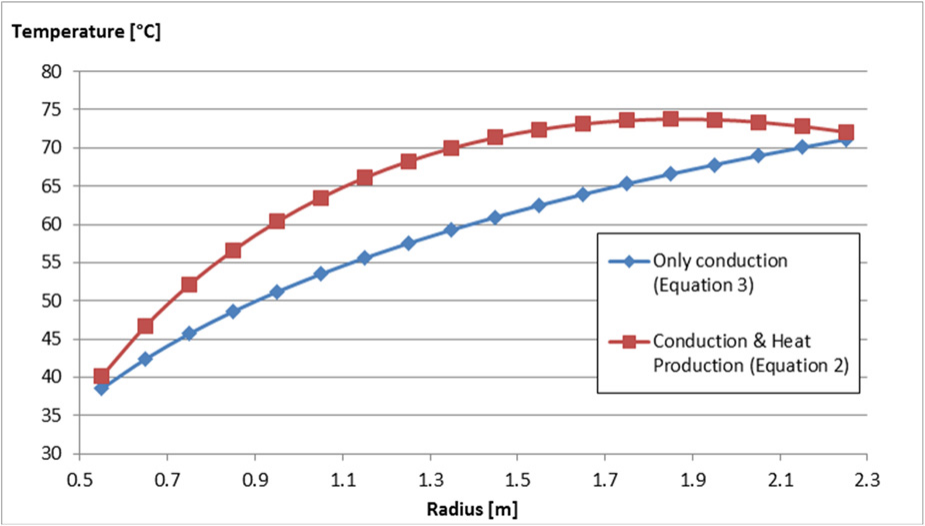
Estimated temperature profiles inside the dunghill at Farm A on 4th November 2009. Cases for Q_prod_ = 0 (only conduction, Equation 3) and for Q_prod_ = 50 W/m^3^ and λ = 2 W/m∙K (conduction and heat production, Equation 2) are shown. The λ value of wet soil is taken [20], the value for Q_prod_ was estimated based on heat transfer calculations using the outdoor air temperature on 4^th^ November 2009.

Examples of temperature data obtained from the measurements and calculated as a result of the heat transfer models of Equations 2 and 3 are shown in Fig. 4. Depending on the values of ${\dot{Q}}_{prod}^{'''}$ and *λ*, the temperature values from Equation 2 may vary, but the general trend remains unaltered.

It is clear from Fig. 4 that the case which includes heat production inside the dung hill shows higher temperature values across the whole range, when compared to the case with only heat conduction. However, the choice of the parameters ${\dot{Q}}_{prod}^{'''}$ and *λ* has a large impact on calculated temperature profiles, and thereby on the survival rates of *C*. *burnetii*. In order to mitigate the risk of overestimating the amount of bacteria that did not survive, Equation 3 was used for all calculations.

For the segmented parts 6–23, results of the temperature profiles, determined reduction percentages of *C*. *burnetii* based on heat resistance of the bacterium in milk [21], and percentages of the volume of the dunghill of every segmented part, are presented in Table 1.

**Table 1:** Estimated temperature profiles in 18 segmented parts of dunghill A.

	Temperature [°C]^b^				Longest consecutive period above a certain temperature [°C]^c^			Reduction[%]^d^	% of the volume of the dunghill (cumulative)^f^
Half ring	<30	30–40	40–50	≥50	T (days)	Max	Average		
1^a^									0.16
2^a^									0.48 (0.64)
3^a^									0.8 (1.44)
4^a^									1.12 (2.56)
5^a^									1.44 (4)
6	36	46	15	0	>40(15)	43	42	^e^	1.76 (5.76)
7	26	47	24	0	>40(17)	44	43	^e^	2.08 (7.84)
8	21	44	32	0	>40(32)	46	44	^e^	2.4 (10.24)
9	17	42	38	0	>40(38)	47	45	^e^	2.72 (12.96)
10	14	39	40	4	>40(44)	51	46	^e^	3.04 (16)
11	10	39	40	8	53(3)	53	53	100	3.36 (19.36)
12	6	38	33	20	≥55(3)	56	55	100	3.68 (23.04)
13	5	36	31	25	≥56(4)	58	57	100	4 (27.04)
14	3	33	33	28	≥55(8)	59	58	100	4.32 (31.36)
15	1	35	30	31	≥57(7)	61	59	100	4.64 (36)
16	1	34	30	32	≥55(10)	62	60	100	4.96 (40.96)
17	1	31	31	34	≥55(11)	64	60	100	5.28 (46.24)
18	1	26	35	35	≥56(11)	65	62	100	5.6 (51.84)
19	1	24	34	38	≥58(11)	67	63	100	5.92 (57.76)
20	1	23	33	40	≥56(12)	68	64	100	6.24 (64)
21	1	23	32	41	≥57(12)	69	65	100	6.56 (70.56)
22	1	22	31	43	≥58(12)	70	66	100	6.88 (77.44)
23	1	20	31	45	≥59(12)	71	67	100	7.2 (84.64)
24^a^									7.52 (92.16)
25^a^									7.84 (100)

^a^Temperature profiles in the segmented parts 1, 2, 3, 4, 5, 24 and 25 fell outside the scope of the two measurement locations in the dunghill (see Fig. 5). These are therefore outside the range of validity of the temperature profile model.

^b^For each segmented part, the number of days that the estimated temperature in the dunghill fell within a certain temperature interval during the 97 days of the experiment is presented.

^c^The combination of the minimum daily temperature (T) with the longest consecutive time interval (days) that could achieve the maximum reduction percentage. In all cases, the highest temperature fell within this period. For the longest consecutive time period also the maximum and the average temperature are determined.

^d^Estimated reduction percentage of *C*. *burnetii* in the dunghill according to comparison with described decimal reduction time (DRT) in milk, as described by Enright et al. [21] and extrapolated using Equation 4.

^e^For the segmented parts 6–10, the reduction percentage of *C*. *burnetii* could not be quantified based on the calculated temperature profiles. Reduction percentages in these segmented parts are less than 100% when compared to DRT of *C*. *burnetii* in milk [21]. Nevertheless, based on DRT in goat manure (see Table 3), survival of *C*. *burnetii* is just above 3 hours at a temperature of 40 degree Celsius. Therefore, total reduction of *C*. *burnetii* in the segmented parts 6–10 might also be possible.

^f^For each segmented part, its contribution (%) to the total volume of the dunghill is presented. Also, the cumulative percentage is presented.

**Fig 5:**
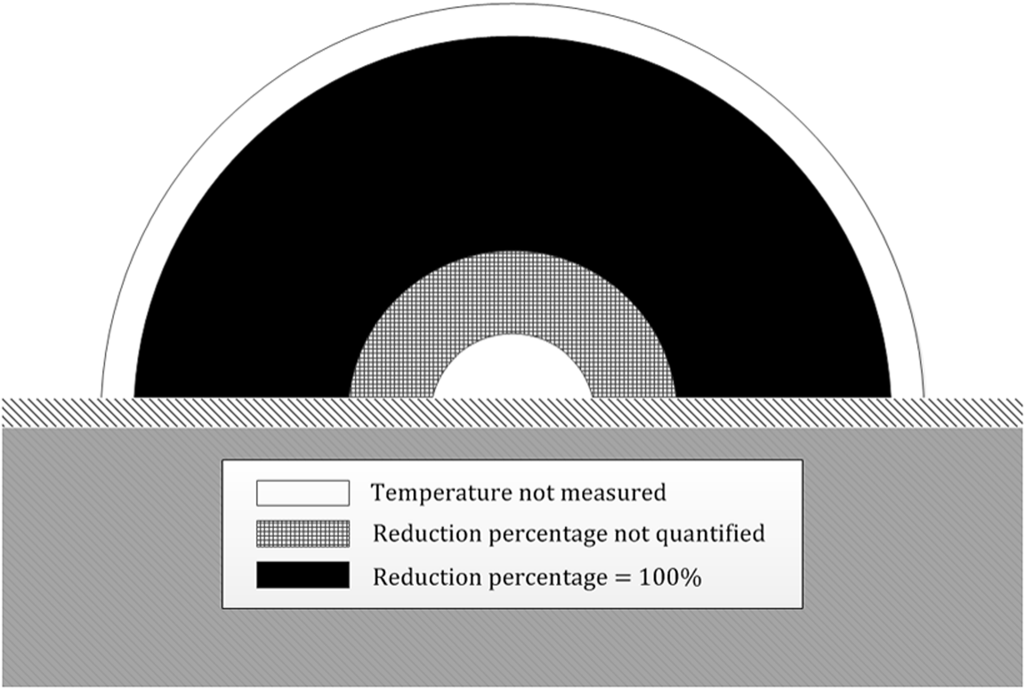
Cross-section of the dunghill. Cross-section of the dunghill with the different layers, for which estimated reduction percentage of C. burnetii according to comparison with described decimal reduction time (DRT) in milk, as described by Enright et al. [21] and extrapolated using Equation 4, are described.

### *Coxiella burnetii* DNA in manure samples

In total, 46 samples were obtained, 22 from farm A and 24 from farm B. Manure samples were categorized into manure location, deep litter stable or dunghill, respectively (Table 2). *C*. *burnetii* DNA was found in manure obtained at all depths of deep litter stables as well as from both dunghills during the whole sampling period. On Farm A, the number of *C*. *burnetii* per gram manure was between 10^3^ and 10^5^. On farm B, the number of *C*. *burnetii* per gram manure was between 10^4^ and 10^7^. The standard deviation ranged between 10^2^ and 10^7^. Due to the presence of multiple copies of the *IS1111* target within the *C*. *burnetii* genome [25, 26], amplification of this target is expected to occur before amplification of the single-copy target *com1*. This was reflected in our data, where for samples showing positive results for both targets *com1* and *IS1111*, Cq values of *IS1111* were consistently lower than those of *com1*. Therefore, positive samples were categorized into two classes with increasing *C*. *burnetii* DNA content: (1) *IS1111* positive and (2) positive for both *IS1111* and *com1*. A number of manure samples showed severe qPCR inhibition in undiluted, and sometimes ten-fold diluted DNA samples. This resulted in the absence of a positive signal for internal control target *cry1*, or amplification curves that showed reduced amplification efficiencies. Samples with no signal for the internal control *cry1* and *C*. *burnetii* targets *IS1111* and *com1* are categorized as ‘not determined’. For quantification purposes, differences between Cq values for internal control target *cry1*, obtained from samples and positive controls, were used to correct for qPCR inhibition effects where possible.

**Table 2:** *Coxiella burnetii* PCR results in manure from two dairy goat farms.

Farm	Manure location	*IS1111*	*IS1111 + com1*	Negative	Not determined
A	Dunghill	1	8		9
	Deep litter stable	2	4		
B	Dunghill	10	3	1	5
	Deep litter stable		2	1	

Number of *C*. *burnetii* positive samples categorized in manure location per farm. The category ‘Not determined’ reflects samples for which no signals were observed in the internal control, or *C*. *burnetii* targets.

### Culture of *Coxiella burnetii* in naïve and spiked goat manure samples

In none of the *C*. *burnetii* PCR positive goat manure samples from both farms, we were able to culture *C*. *burnetii*. In order to exclude technical problems, *C*. *burnetii* was cultured from *C*. *burnetii* spiked solutions of goat manure samples (positive results of the positive control) taken from the floor in the deep litter stable. Both in immediate culture as in samples after 48 hour incubation, *C*. *burnetii* could be cultured. Therefore, technical culture problems were excluded.

### Calculated decimal reduction time

Results of the calculated decimal reduction time (DRT) of the Nine Mile (NM) RSA 493 reference strain of *C*. *burnetii* under experimental circumstances are presented in Table 3. DRT in milk [21] was longer than we found in the measurements within goat manure, ammonia, urea and PBS. Extrapolation to a temperature of 40°C indicated that the DRT of the NM reference strain of *C*. *burnetii* in goat manure was just above 3 hours.

**Table 3:** Decimal reduction time (in seconds) of the Nine Mile reference strain of *Coxiella burnetii* at different temperatures in different matrices.

	DRT in seconds (minutes; hours (when the number of hours stayed above 0,1))				
	NM in PBS	NM in 1.8% ammonia	NM in 1.8% urea	NM in manure from deep litter stable	NM in milk (Enright et al., 1957)
Temperature (t) (°C)	10^^^(-0.1139t+8.7138)	10^^^(-0.1355t+10.383)	10^^^(-0.1222t+9.4457)	10^^^(-0.0996t+8.0317)	10^^^(-0.2253t+17,3307)
40	14381 (240; 4)^a^	918333 (15306; 255)^a^	36116 (602; 10)^a^	11161 (186; 3.1)^a^	208305147 (3471752; 57863)^a^
50	1044^a^	4055 (68; 1,1)^a^	2166 (36; 0,6)^a^	1126 (19; 0.3) ^a^	1163322 (19389; 323)^a^
60	66,0	113,3	123,7	113,7	6497 (108; 1,8)^a^
65	30,0	102,2	40,0	36,1	486 (8; 0,1) ^b^
70	3,3	3,8	4,6	11,5	36
72	4,3	5,2	6,3	7,3	13

DRT, decimal reduction time; NM, Nine Mile reference strain of *C*. *burnetii*; PBS, Phosphate Buffer Saline;

^a^Extrapolated DRT results;

^b^Intrapolated DRT result.

## Discussion

During the human Q fever outbreak (2007–2010) in the Netherlands, which occurred primarily in the south-eastern part of the country, manure from dairy goat farms has been transported to several other parts of the country. We found no increased incidence of human Q fever related to distribution of manure originating from dairy goat farms with confirmed abortion waves caused by *C*. *burnetii*. Several studies have shown that living within a radius of five km from an infected farm was an independent risk factor for acquiring human Q fever [5, 8–12]. In these studies, distributions of manure from an infected farm with small ruminants were not described as risk factor for human Q fever, which is now supported by our study as well. In another Dutch study, distribution of goat manure was actually linked to human Q fever cases [27]. However, these results are difficult to compare with our results for several reasons. Hermans et al. [27] did not include control herds, did not only include goat farms with abortion waves caused by *C*. *burnetii*, but also included herds that only tested PCR positive in the BTM surveillance program, and included distributions of manure to an area within a radius of five and ten km around infected farms. We believe that within a small geographical area it is not possible to determine whether clusters of human Q fever patients are caused by transmission from land-applied goat manure or by airborne transmission from infected herds. Based on our results and bias in the study design of Hermans et al. [27], we find it highly unlikely that land-applied goat manure played an important role as a source of human Q fever.

Although a large amount of *C*. *burnetii* DNA was present in manure samples from both participating farms with a recent history of *C*. *burnetii* related abortion, we were not able to culture *C*. *burnetii* from any of these manure samples. We were able to culture *C*. *burnetii* from spiked manure samples, demonstrating that technically it was possible to isolate *C*. *burnetii* from a complex matrix like manure. Although serial passages in experimental hosts is the most accurate procedure for determining the presence of small numbers of viable *C*. *burnetii* [21], our negative culture results suggest that no or only low numbers of viable *C*. *burnetii* were present in the manure samples.

The results of this study show that temperatures in the core and shell of the dunghills on farm A and B were above 40°C for at least ten consecutive days. Temperature measurements showed a higher temperature in the shell compared to the core. This difference probably is a result of the fact that successful composting is influenced by the availability of oxygen, and compulsory covering of a dunghill can therefore negatively influence the composting process. Temperature profiles calculated for farm A indicate a reduction in numbers of *C*. *burnetii* in the segmented parts 11–23 of 100%. In the segmented parts 6–10, temperatures were not high enough for a certain consecutive period of time to be certain that a total reduction of *C*. *burnetii* occurred. Temperature profiles of the segmented parts 1–5 and 24–25 fell outside the two measuring points (Fig. 5), and we chose not to incorporate them and consequently neither could a reduction percentage be determined. Segmented parts 1–5, 6–10, 11–23, and 24–25 represent 4, 12, 68.6 and 15.4 per cent of the total volume of the dunghill, respectively. The segmented parts for which temperature profiles could be determined (6–23) represent about 81 per cent of the volume of the dunghill. Because of a lack of measuring points in the segmented parts 1–5 and 24–25, temperature profiles and therefore reduction percentages of *C*. *burnetii* could not be determined for about 19 percent of the volume of the dunghill. Based on these temperature profiles, and the DRT according to Enright et al. [21], it can be concluded that in at least 85 per cent (68.6/80.6) of the volume of segmented parts 6–23 probably no *C*. *burnetii* could have survived the composting process.

Heat resistance of *C*. *burnetii* has been validated in infection studies in guinea pigs [21]. In that study, two time-temperature combinations were finally found to be effective for pasteurization purposes and have subsequently been universally recognized: 30 minutes at 62.8°C (degrees Celsius, 145 degrees Fahrenheit) or 15 seconds at 71.7°C (161 degrees Fahrenheit) [21]. These recommendations were simplified as: 30 minutes at 63°C or 15 seconds at 72°C, thus providing an extra safety margin. Assuming the 10log survival curve is a straight line, this would achieve eight decimal reductions [28]. For other matrices than milk, the decimal reduction time (DRT) of the *C*. *burnetii* Nine Mile (NM) RSA 493 reference strain has not been described before. In this study, DRT measured under experimental conditions appeared to be shorter in PBS, ammonia, urea, and goat manure, compared to the DRT of *C*. *burnetii* in milk [21]. Extrapolation of these results to a temperature of 40°C, results in a DRT of the NM reference strain of *C*. *burnetii* in goat manure of just above 3 hours. In that case, survival of *C*. *burnetii* in the segmented parts 6–10 of the dunghill, based on the estimated temperature profiles, is very unlikely. A shorter DRT of the NM RSA 493 reference strain of *C*. *burnetii* in manure compared to milk can be caused by biological, physical, and chemical variables that may influence survival of bacterial pathogens in manure [29]. Survival of several food borne pathogens such as *Escherichia coli* O157:H7 and *Salmonella enteritidis* has been investigated, and in properly composted manure microbial contamination seems to be minimized [30]. Although, compared to pathogens like *Salmonella* spp., spore-forming bacteria seem to be able to survive pasteurization for a longer period [31]. Sharma et al. [32] showed that despite reduction of antimicrobially resistant *E*. *coli*, antimicrobially resistant genes from these bacteria could be detected and therefore it was discussed whether using PCR should be preferred over cultivation-based methods for rapid identification of composting effectiveness.

As a precautionary principle, we applied a worst case scenario in all our calculations for the temperature profiles in the 25 segmented parts in which we mathematically segmented the dunghill on farm A. This means that we assumed that only heat conduction and no heat production in the dunghill took place. Furthermore, we did not perform extrapolation of temperature profiles outside the two measuring points, and we compared the temperature profiles to the higher DRT of *C*. *burnetii* in milk rather than comparing it with the lower DRT which we experimentally measured in goat manure. Consequently, it is very likely that the percentage of surviving *C*. *burnetii* is lower in reality than the values presented in this study. In a follow-up study we would recommend to extend the number of temperature measuring points to at least five in order to be able to estimate temperature profiles more accurately, without extrapolation, for all 25 segmented parts in a dunghill. Under such conditions, it would also be possible to determine heat conduction as well as heat production in composting dunghills, making an even more accurate estimation possible. The five recommended measurement locations are: core, shell (dung hill top), shell (at concrete floor), halfway between shell and core (vertically), and halfway between shell and core (horizontally). This follow-up would not only be of interest for *C*. *burnetii*, but also for determining survival possibilities in a dunghill for other pathogens, especially those with zoonotic potential. Additionally, it would be of interest to perform similar studies in different countries with different types of piles of manure.

In conclusion, several studies have suggested that manure from ruminants played an important role in the transmission of *C*. *burnetii* to humans [33–35]. Arricau-Bouvery and Rodolakis [1] stated that manure from infected herds should be covered and composted or treated with lime or calcium cyanamide 0.4% before being spread on the field, and spreading should never be performed under windy circumstances. In our study, no relation could be found between distributions of goat manure and incidence of human Q fever. The same applies for epidemiological risk factor studies, where manure was not found to be a risk factor for human Q fever. Although a large amount of *C*. *burnetii* DNA was present in manure samples from both farms, we were not able to culture *C*. *burnetii*. Even if viable *C*. *burnetii* had been present, composting would have resulted in a large reduction, taking into account core and shell time and temperature profiles, heat resistance of *C*. *burnetii* as described by Enright et al. [21], and the decimal reduction time of the Nine Mile RSA 493 reference strain of *C*. *burnetii* in manure determined in this study. Thus, land-applied goat manure probably played a minor role in the transmission of *C*. *burnetii* to humans in the 2007–2010 Dutch Q fever outbreak, possibly partly due to a proper composting process.
